# Measuring the criticality of the ‘magic condition’ for a beam-expanding monochromator

**DOI:** 10.1107/S1600577516012650

**Published:** 2016-10-06

**Authors:** Mercedes Martinson, Dean Chapman

**Affiliations:** a Physics and Engineering Physics, University of Saskatchewan, 116 Science Place, Rm 163, Saskatoon, Saskatchewan, Canada S7N 5E2; b Canadian Light Source, 44 Innovation Boulevard, Saskatoon, Saskatchewan, Canada S7N 2V3

**Keywords:** magic condition, bent Laue double-crystal monochromator, geometric and polychromatic focus

## Abstract

It has been established that for cylindrically bent crystals the optimal beam characteristics occur when the geometric and single-ray foci are matched. In the beam-expanding monochromator developed for the BioMedical Imaging and Therapy beamlines at the Canadian Light Source, it was unclear how critical this ‘magic condition’ was for preserving the transverse coherence of the beam. A study was conducted to determine whether misalignments away from the ideal conditions would severely affect the transverse coherence of the beam, thereby limiting phase-based imaging techniques. The results were that the magic condition has enough flexibility to accommodate deviations of about ±1° or ±5 keV.

## Abstract 2

The effect of minor mismatch between the geometric and single-ray foci for a cylindrically bent Laue double-crystal monochromator is examined and found to be less detrimental than previously believed. Even without exact matching, the transverse coherence of the X-ray beam is not deteriorated by the system, enabling the phase-based imaging techniques critical to modern biomedical imaging experiments.

## 1. Introduction   

**Figure 1:**
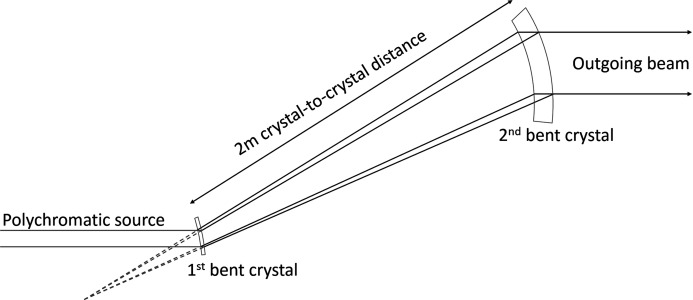
Experimental setup.

A double bent Laue beam-expanding monochromator (Fig. 1 ▸) has been designed for the BioMedical Imaging and Therapy (BMIT) beamlines at the Canadian Light Source. During our earlier work (Martinson *et al.*, 2014 ▸), significant beam blurring in the vertical direction (corresponding to horizontally oriented object edges) was believed to be caused by a mismatch between the single-ray and geometric focus types. A key improvement in the design was the preservation of the transverse coherence of the beam (Martinson *et al.*, 2015 ▸), which allows phase-sensitive imaging techniques to be performed with a large field of view. This was achieved by matching the two focus types (single-ray focus, 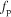, and geometric focus, 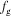) in the first crystal to each other and to the geometric focus of the second crystal. At the time it was unclear how sensitive the system was to deviations from this ‘magic condition’.

The single-ray focus equation (Martinson *et al.*, 2015 ▸) is
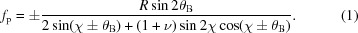



The geometric focus equation (Schulze *et al.*, 1998 ▸) is 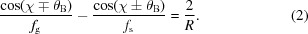



For this study, the magic condition was determined from the first crystal in the expander system, which uses a (3,1,1)-type reflection on a (5,1,1) silicon wafer (producing an asymmetry angle of 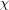 = 3.33°), at a bend radius 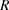 = 0.5 m and a source-to-crystal distance 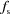 = 22 m for the BMIT bend-magnet beamline. By setting 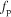 = 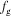 and assuming a Poisson ration of 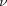 = 0.22, the magic condition is determined numerically to occur at a Bragg angle 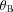 = 7.55°.

## 2. Experimental procedure   

**Figure 2:**
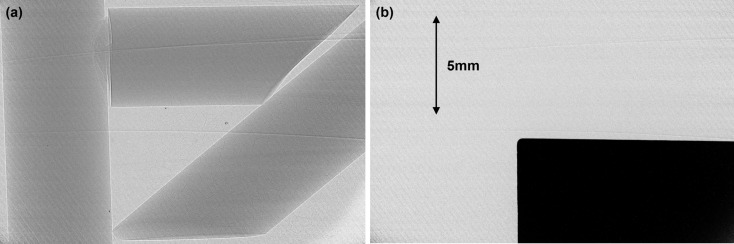
Sample (*a*) phase and (*b*) knife-edge images.

The beam-expanding system was set up as shown by Martinson *et al.* (2014 ▸) with the geometric focus of the second crystal matched to that of the first crystal. The bend radii of the first and second crystals were 0.5 m and 5 m, respectively, producing an expansion factor of approximately 10, with a crystal-to-crystal distance of approximately 2 m. Using a Hamamatsu detector [AA-60 beam monitor coupled to a C9300-124 CCD camera resulting in a field of view of 31.08 mm (H) × 23.31 mm (V) and pixel size 8.75 µm] and object-to-detector distance of 134 cm, images of a knife-edge (tungsten bar) and phase object (Lucite rod) were captured through Bragg angles ranging ±1° from the magic condition (see Fig. 2 ▸). At each Bragg angle the two crystals were carefully aligned (*i.e.* diffraction planes and geometric foci were matched) to optimize beam intensity.

## 3. Analysis   

Both vertically and horizontally oriented edges were analysed for each test object and Bragg angle using the procedure given by Martinson *et al.* (2015 ▸). The phase peak width was measured using a pseudo-Gaussian fit to measure the distance (in pixels) between inflection points in the plot profile. The knife-edge width was measured as the FWHM (in pixels) of a Gaussian fit to the derivative of the plot profile. To account for misalignment between the samples’ edges and the detector pixel lines, the peak width was minimized with respect to the rotation angle of cropped subsections (100 pixels wide across the edge and varying between 5, 10 and 25 pixels wide along the edge). The final width measurement for each edge was then taken as the mean of these minimized widths, with an uncertainty equal to half the difference of the largest and smallest. The measurement results are presented in Table 1 ▸, where the horizontal and vertical labels refer to the orientation of the object’s edge relative to physical space and are perpendicular to the vertical and horizontal diffraction planes, respectively.

## 4. Discussion   

In the knife-edge images the vertical and horizontal edges all agreed within experimental uncertainty at each Bragg angle, differing by at most 0.72%. The expectation of the vertical edges width being equal at all Bragg angles was very nearly realised, with the 8.55° sample failing equality by only 0.004 pixels.

In the phase images these results do not hold. It is noted that the signal-to-noise ratio in the phase images was very poor due to the significant noise of the images and low relative signal of the phase fringe. This likely caused poor fits in the procedure, resulting in unreliable results. Nonetheless, the measurements are presented for completeness.

## 5. Conclusion   

Overall, the small difference in edge width as the angle moves away from the magic condition, along with visual inspection of the phase images at all Bragg angles measured, indicates that strict magic conditions are not required for the purposes of medical imaging. This is advantageous for applications that require specific energies (*e.g.* *K*-edge subtraction) or beamline configurations (*e.g.* fixed Bragg angle due to apparatus construction). This creates flexibility in the system, as a separate set of crystals with specific asymmetry angles is not required in order to change energies. While ideal matching may be required for certain microfocusing applications of bent Laue double-crystal monochromators, it would appear that, as long as both crystals are in the upper sign geometry [*i.e.* the tilt angle of crystal is 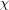 + 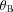 instead of the 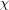 − 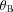 tilt that is now believed to be the primary cause of the beam blurring observed in our earlier work (Martinson *et al.*, 2014 ▸)], the system will produce a suitable beam for biomedical imaging with phase contrast techniques.

## Figures and Tables

**Table 1:** Peak widths of phase and knife-edge images as a function of Bragg angle

		Knife object; fringe width in pixels			Phase object; edge width in pixels		
Bragg angle (°)	Energy (keV)	Vertical	Horizontal	(|*V* − *H*|/*V*) × 100%	Vertical	Horizontal	(|*V* − *H*|/*V*) × 100%
6.55	33.2	3.058 ± 0.010	3.042 ± 0.008	0.52%	3.316 ± 0.039	5.119 ± 1.162	54%
7.05	30.9	3.059 ± 0.009	3.059 ± 0.007	0.00%	4.501 ± 0.085	4.982 ± 2.591	11%
7.30	29.8	3.076 ± 0.020	3.063 ± 0.010	0.42%	4.718 ± 0.241	6.414 ± 2.210	36%
7.55	28.8	3.073 ± 0.020	3.063 ± 0.010	0.33%	4.761 ± 0.010	4.505 ± 0.621	5.4%
7.80	27.9	3.059 ± 0.012	3.037 ± 0.010	0.72%	3.621 ± 0.003	7.092 ± 2.624	96%
8.05	27.0	3.063 ± 0.010	3.054 ± 0.005	0.29%	3.948 ± 0.190	4.302 ± 0.443	9.0%
8.55	25.5	3.051 ± 0.001	3.058 ± 0.007	0.23%	6.868 ± 0.025	5.114 ± 0.823	26%

## References

[bb1] Martinson, M., Samadi, N., Bassey, B., Gomez, A. & Chapman, D. (2015). *J. Synchrotron Rad.* **22**, 801–806. 10.1107/S1600577515004695 PMC4416688 25931100

[bb2] Martinson, M., Samadi, N., Belev, G., Bassey, B., Lewis, R., Aulakh, G. & Chapman, D. (2014). *J. Synchrotron Rad.* **21**, 479–483. 10.1107/S1600577514003014 PMC3998813 24763635

[bb3] Schulze, C., Lienert, U., Hanfland, M., Lorenzen, M. & Zontone, F. (1998). *J. Synchrotron Rad.* **5**, 77–81. 10.1107/S0909049597014568 16687807

